# Intersectionality of individual and neighborhood‐level adverse social determinants of health in early pregnancy

**DOI:** 10.1002/pmf2.70002

**Published:** 2025-02-27

**Authors:** Jameaka L. Hamilton, William A. Grobman, Jiqiang Wu, Lynn M. Yee, David Haas, Becky Mcneil, Brian Mercer, Hyagriv Simhan, Uma Reddy, Robert M. Silver, Samuel Parry, George Saade, Jun Wu, Courtney D. Lynch, Kartik K. Venkatesh

**Affiliations:** ^1^ Department of Obstetrics and Gynecology The Ohio State University Columbus Ohio USA; ^2^ Department of Obstetrics and Gynecology Northwestern University Chicago Illinois USA; ^3^ Department of Internal Medicine The Ohio State University Columbus Ohio USA; ^4^ RTI International Durham North Carolina USA; ^5^ Department of Obstetrics and Gynecology Case Western Reserve University Cleveland Ohio USA; ^6^ Department of Obstetrics and Gynecology University of Pittsburgh Pittsburgh Pennsylvania USA; ^7^ Department of Obstetrics and Gynecology Columbia University New York New York USA; ^8^ Department of Obstetrics and Gynecology University of Utah Salt Lake City Utah USA; ^9^ Department of Obstetrics and Gynecology University of Pennsylvania Philadelphia Pennsylvania USA; ^10^ Department of Obstetrics and Gynecology University of Texas Medical Branch Galveston Texas USA; ^11^ Department of Environmental and Occupational Health University of California, Irvine Orange California USA; ^12^ Present address: Department of Obstetrics and Gynecology Brown University Providence RI USA

**Keywords:** individual, neighborhood, pregnancy, social determinants of health

## Abstract

**Introduction:**

Individual‐ and neighborhood‐level social determinants of health (SDOH) have been assessed separately in pregnancy, but their relationship to one another remains uncertain. We investigated the intersectionality of three neighborhood‐level SDOH measures with three individual‐level SDOH measures. This was done to examine the concomitant experiences of multiple SDOH in pregnancy.

**Methods:**

A secondary analysis of data from the Nulliparous Pregnancy Outcomes Study: Monitoring Mothers‐To‐Be. We assessed three neighborhood‐level SDOH measures using geocoded participant home addresses in the first trimester at the census‐tract level: (1) high socioeconomic disadvantage (in tertiles) by the 2015 Area Deprivation Index, (2) inadequate food access by the USDA *Food Access Research Atlas*, and (3) low walkability by the EPA National Walkability Score. We assessed three individual‐level SDOH measures: low household income, lower educational attainment, and Medicaid insurance. We examined the combinations of these three neighborhood SDOH and three individual SDOH measures by graphical visualization and using statistical tests to assess overall differences in the distribution of these measures.

**Results:**

Of 9588 nulliparous individuals, adverse neighborhood‐level SDOH [high socioeconomic disadvantage (28%), inadequate food access (24%), and low walkability (66%)] and adverse individual‐level SDOH [low household income (19%), lower educational attainment (23%), and Medicaid insurance (33%)] were common in early pregnancy. Six percent of individuals lived in a community with all three adverse neighborhood‐level SDOH measures. Of those living in a community with at least two neighborhood‐level SDOH measures, 23% lived in areas with inadequate food access and low walkability, 19% with high socioeconomic disadvantage and low walkability, and 1% with high socioeconomic disadvantage and inadequate food access. Overall, 23% lived in a community with no adverse neighborhood‐level SDOH, and among this group, 88% had no adverse individual‐level SDOH. There were significant differences in adverse individual‐level SDOH based on whether individuals lived in a community with all three adverse neighborhood‐level measures [low household income (39%), lower educational attainment (44%), Medicaid (55%)], any two measures [low household income (22%), lower educational attainment (27%), Medicaid (37%)], or only one measure [low household income (14%), lower educational attainment (17%), Medicaid (27%)] (*p* < 0.001 for all).

**Conclusion:**

Among nulliparous individuals in early pregnancy, the frequency of adverse individual‐level SDOH was generally higher when they lived in communities with more adverse neighborhood‐level SDOH. Future approaches that identify and classify the multifaceted and multilevel nature of structural determinants as they relate to pregnancy outcomes are needed.

## 1 INTRODUCTION

The impact of adverse social determinants of health (SDOH) on adverse pregnancy outcomes (APOs) is an area of increasing clinical, research, and policy focus given significant and persistent disparities in maternal and infant morbidity and mortality in the United States (US) [1, 2]. The World Health Organization (WHO) and the US Healthy People 2030 define SDOH as “non‐medical factors that influence health outcomes” which can include “conditions in which people are born, grow, work, live, and age” [3, 4]. SDOH is a multidimensional construct that can be measured at an individual level, a broader community level, or neighborhood level [5].

Individual‐level SDOH (iSDOH) that are associated with poor pregnancy outcomes include lower income, experiences of racial and ethnic discrimination, limited access to educational opportunities leading to low educational attainment, poor housing quality, unemployed status, and lack of nutritious food and opportunities for physical activity. At the community level, the neighborhoods where individuals reside shape environmental exposures, access to resources, and opportunities [3, 4]. In comparison to iSDOH, neighborhood‐level SDOH (nSDOH) have been less characterized during pregnancy.

Adverse nSDOH, which are often derived from geocoded residential or work addresses, include multidimensional constructs, such as the Area Deprivation Index (ADI) or Social Vulnerability Index (SVI), or more specific constructs, such as those that quantify access to food and walkable spaces [6, 7, 8, 9, 10, 11, 12]. nSDOH that are associated with APOs include less access to public transportation, residential racial and ethnic segregation, greater distance to healthcare facilities, and less access to healthy foods and spaces to exercise [4, 5]. Emerging data support that each of these measures is associated with APOs, such as glycemic control for pregnant individuals with pre‐gestational diabetes [13, 14, 15], postpartum readmission [16], and rates of vaccination in pregnancy [17].

The extent to which nSDOH measures overlap with iSDOH measures remains to be studied, in particular in pregnancy [18]. As highlighted by recent professional society recommendations, understanding such relationships is critical for future epidemiologic investigation to identify patterns of iSDOH and nSDOH that are associated with the highest risk of APOs and to develop effective multilevel interventions that best address these underlying factors [19, 20].

We examined the intersectionality of three nSDOH measures (socioeconomic disadvantage, food access, and walkability) and three iSDOH measures (household income, educational attainment, and Medicaid insurance status) to model the relationship of different SDOH in nulliparous individuals in early pregnancy.

## 2 MATERIALS AND METHODS

### 2.1 Study setting

This is a cross‐sectional analysis using data from Nulliparous Pregnancy Outcomes Study: Monitoring Mothers‐To‐Be (nuMoM2b), a prospective cohort that was designed to evaluate maternal and environmental contributors to poor birth outcomes. In this cohort, individuals were enrolled at eight US medical centers from October 2010 to September 2013 (ClinicalTrials.gov NCT01322529). Full details of this study have been described previously [21]. Institutional Review Board approval was obtained at each nuMoM2b study site, and all participants provided written informed consent prior to participation.

### 2.2 Study participants

Nulliparous individuals met inclusion criteria for study enrollment if they had no prior history of delivery at 20 weeks’ gestation or later, had a viable singleton pregnancy with estimated gestational age between 6 weeks 0 days to 13 weeks 6 days at enrollment, and intended to deliver at a participating site hospital. Individuals were excluded from the study if less than 13 years old, had a history of three or more pregnancy losses, had a current pregnancy conceived via donor oocyte, planned a pregnancy termination, had a likely lethal fetal malformation, had a fetal aneuploidy, were previously enrolled in the study, or had an inability to provide informed consent. In addition, individuals were excluded from the current analysis if they did not have a residential US address at the time of enrollment that could be geocoded.

### 2.3 Study measures

The primary residential addresses of participants in the nuMoM2b study were collected via structured interview at the first study visit (6–13 weeks’ gestation). Addresses were subsequently geocoded using ArcGIS (www.arcgis.com; Esri, Redlands, CA), and linked to census‐tract level nSDOH. A census tract is a statistical subdivision of a county with relatively permanent boundaries that allows comparison of an area between different census years; census tracts generally have a population size between 1200 and 8000 people [22].

Three iSDOH measures that are commonly recorded in the electronic health record and used in the context of care delivery and pregnancy research were evaluated: (1) low household income, defined as less than 100% of the 2015 US Federal Poverty Level (FPL); (2) lower educational attainment, defined as having a high school education or less; and (3) Medicaid or public health insurance status.

Three nSDOH measures that have increasingly been studied in pregnancy were evaluated: (1) living in a neighborhood with high socioeconomic disadvantage, (2) inadequate food access, and (3) low walkability. Neighborhood socioeconomic disadvantage was assessed using the 2015 version of the Area Deprivation Index (available at https://www.neighborhoodatlas.medicine.wisc.edu/). The ADI uses 17 US Census indicators across income, education, employment, and housing quality domains to generate a composite score which is converted to a percentile from 0 to 100 to rank disadvantaged communities nationally [6, 7, 23]. The ADI is analyzed based on national tertiles from low (tertile 1, [T1]) to high (tertile 3, [T3]), with the highest tertile being associated with communities experiencing the greatest socioeconomic disadvantage [16, 17].

Neighborhood food access was defined using the *Food Access Research Atlas* of the United States Department of Agriculture (USDA) Economic Research Service, which provides a spatial overview of neighborhood‐level food access, with inadequate access defined as the lack of consistent and sustainable means to access safe, culturally acceptable, and adequately nutritious foods in a specific geographic location, based on indicators of food access by income and supermarket accessibility [24, 25]. Those living in a community with inadequate food access were identified if both low income and low food access criteria were met: a census‐tract level in which at least 20% of the population had a median family income ≤ 80% of the metropolitan area or state median income, and a number of at least 500 and proportion of at least 33% of individuals living > 1 mile (urban setting) or > 10 miles (rural setting) from the nearest grocery store, respectively [8, 9, 26].

Neighborhood walkability was defined using the Environmental Protection Agency (EPA) National Walkability Index based on equally weighted components of “intersection density” (higher density correlating with more frequent walking trips), “proximity to transit stops” (shorter transit stop distance correlating with more frequent walking trips), “employment mix” (increased employment type diversity [retail, office, industrial] correlating with more frequent walking trips), and “employment and household mix” (more diverse employment types plus many occupied housing units correlate with more walk trips) [10, 11]. Index scores ranged from 1 to 20, with lower scores indicating that a lower proportion of people living in a community use walking as a mode of transportation [10]. Neighborhoods considered to have low walkability, based on EPA recommendations, were those with walkable scores < 15.26 [11].

### 2.4 Statistical analysis

We first graphically examined the relationship between the three nSDOH measures by using a Venn diagram to visually assess the extent of overlap between neighborhood socioeconomic disadvantage, food access, and walkability. We then examined the relationship of each of above three nSDOH measures with the three iSDOH measures, namely household income, educational attainment, and Medicaid insurance status, both in graphical and tabular format. We examined differences in the distribution of iSDOH measures by nSDOH measures using overall chi‐square tests. All statistical analyses were performed using R statistical software version 4.2.0 (R Foundation for Statistical Computing). A *p* value < 0.05 based on a two‐tailed test was considered statistically significant.

## 3 RESULTS

Of the 10,038 individuals in the cohort, 9588 (95%) were included in this secondary analysis based on availability of a residential address which could be geocoded and linked to the assessed nSDOH measures. Sociodemographic and clinical characteristics of the study population overall and when stratified by the three nSDOH measures (area deprivation, food access, and walkability) are shown in Table 1. The mean age was 27 years old (SD: 22.0, 31.0), with 13% of individuals self‐identifying as non‐Hispanic Black and 17% as Hispanic.

**TABLE 1:** Sociodemographic and clinical characteristics of the study population overall and by three neighborhood‐level SDOH measures (*N* = 9588).

		Socioeconomic disadvantage			Inadequate food access		Low walkability	
Overall	Tertile 1 (low)	Tertile 2	Tertile 3 (high)	Yes	No	Yes	No
Neighborhood‐level exposure, % (IQR)	39.0 (18.0, 71.0)	13.0 (8.0, 19.0)	39.0 (32.0, 47.0)	87.0 (72.0, 95.0)^*^	37.0 (23.0, 59.0)	40.0 (17.0, 76.0)	41.0 (22.0, 73.0)	34.0 (14.0, 69.0)^*^
Age, years, median (IQR)	27.0 (22.0, 31.0)	30.0 (26.0, 33.0)	27.0 (24.0, 31.0)	23.0 (20.0, 28.0)^*^	26.0 (22.0, 30.0)	28.0 (23.0, 31.0)^*^	26.0 (22.0, 30.8)	29.0 (24.0, 32.0)^*^
Medicaid insurance (*N* = 9525)	2653 (27.9)	464 (14.2)	557 (17.7)	1632 (52.3)	564 (24.5)	2089 (28.9)	1954 (31.0)	699 (21.7)
Race and ethnicity								
Asian American	382 (4.0)	220 (6.7)	93 (3.0)	69 (2.2)	50 (2.2)	332 (4.6)	219 (3.4)	163 (5.0)
Black	1298 (13.5)	127 (3.9)	219 (7.0)	952 (30.1)	258 (11.1)	1040 (14.3)	921 (14.5)	377 (11.6)
Hispanic	1603 (16.7)	470 (14.4)	419 (13.3)	714 (22.6)	201 (8.7)	1402 (19.3)	1111 (17.5)	492 (15.2)
Multiracial	488 (5.1)	140 (4.3)	140 (4.4)	208 (6.6)^*^	112 (4.8)	376 (5.2)^*^	311 (4.9)	177 (5.5)^*^
White	5817 (60.7)	2318 (70.8)	2280 (72.4)	1219 (38.6)	1694 (73.2)	4123 (56.7)	3788 (59.7)	2029 (62.7)
Education (*N* = 9581)								
High school or less	730 (7.6)	78 (2.4)	141 (4.5)	511 (16.2)	180 (7.2)	550 (7.6)	550 (8.7)	180 (5.6)
Some college or higher	8851 (92.4)	3196 (97.6)	3009 (95.5)	2646 (83.8)^*^	2315 (92.8)	6716 (92.4)^*^	5794 (91.3)	3057 (94.4)^*^
Tobacco use (*N* = 9578)	1680 (17.5)	409 (12.5)	480 (15.2)	791 (25.0)	484 (20.9)	1196 (16.5)	1191 (18.8)	489 (15.1)
Body mass index, kg/m^2^ (*N* = 9399)								
BMI < 30	7330 (78.0)	2782 (86.2)	2415 (77.6)	2133 (69.7)	1744 (76.0)	5586 (78.6)	4716 (75.9)	2614 (82.1)
BMI ≥ 30	2069 (22.0)	445 (13.8)	698 (22.4)	926 (30.3)^*^	551 (24.0)	1518 (21.4)^*^	1499 (24.1)	570 (17.9)^*^
Household income and size relative to US poverty level (*N* = 7846)								
<130%	5499 (70.1)	2570 (87.5)	2014 (74.2)	915 (41.7)	1303 (68.0)	4196 (70.8)	3397 (66.6)	2102 (76.7)
≥130%	2347 (29.9)	366 (12.5)	701 (25.8)	1280 (58.3)	613 (21.0)	1734 (29.2)	1707 (33.4)	640 (23.3)

*Note*: Data are *n* (%) unless otherwise specified.

* *p* < 0.001 for assessed characteristics above.

With regard to iSDOH, 16% of individuals lived in a household with low income, 8% had lower education attainment, and 28% were Medicaid insured. With regard to nSDOH, 28% of individuals lived in a community with more socioeconomic disadvantage, 24% with lack of adequate food access, and 66% with low walkability. Overall, 23% lived in a community with no adverse nSDOH of whom 88% had no adverse iSDOH.

**FIGURE 1:**
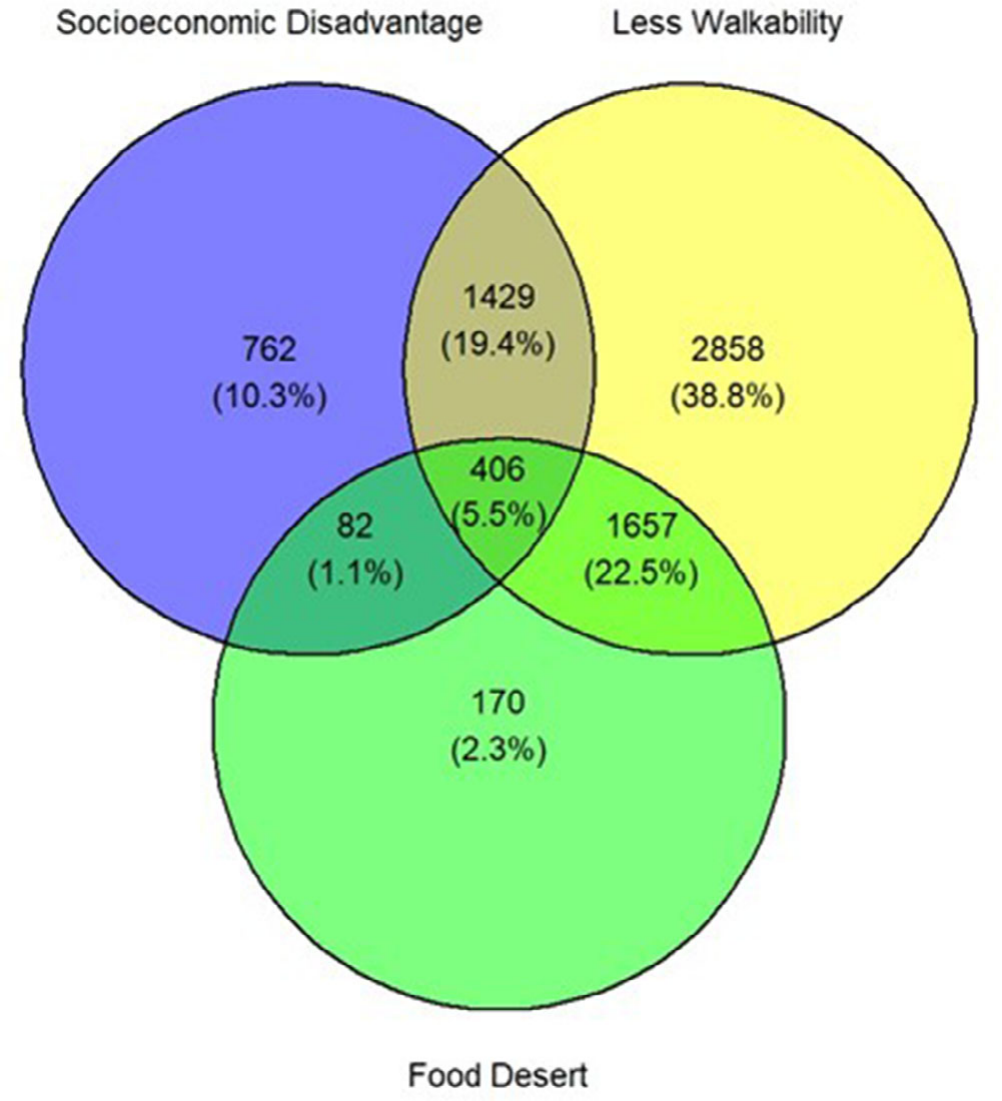
Intersection of adverse neighborhood‐level social determinants of health in Nulliparous individuals in early pregnancy. Venn diagram demonstrating the intersectionality of living in a neighborhood with high socioeconomic disadvantage, inadequate food access, and low walkability in early pregnancy. The total N included individuals with nSDOH data for all three measures (N=7,364).

The overlap in the frequency of the three nSDOH is visually represented in Figure 1 as a Venn diagram. Living in a community with all three adverse nSDOH measures was relatively uncommon (6%), while living in a community with any two adverse nSDOH measures occurred in 43% of individuals. The remaining individuals (51%) lived in a community with one adverse nSDOH: low walkability alone (38%), high socioeconomic disadvantage alone (10%), or inadequate food access alone (2%).

The intersections of the three iSDOH and three nSDOH are presented in Table 2. Of those living in a community with high socioeconomic disadvantage only, 37% of individuals had low household income, 3% had low educational attainment, and 50% had Medicaid insurance. When low walkability was the only adverse nSDOH, 10% of individuals had low household income, 13% had lower educational attainment, and 21% had Medicaid insurance. Conversely, when living in a community with inadequate food access alone, 10% of individuals had low household income, 11% had lower educational attainment, and 15% had Medicaid insurance.

**TABLE 2:** Intersection of individual‐level SDOH among individuals experiencing one, two, or three overlapping neighborhood‐level SDOH (*N* = 9588).

	Individual‐level SDOH					
	Low household income^a^		Lower educational attainment^a^		Medicaid insurance^a^	
	Yes	No	Yes	No	Yes	No
**One neighborhood‐level SDOH**						
Low walkability only	2201 (90.1)	241 (9.9)	2485 (87.0)	371 (13.0)	601 (21.1)	2248 (78.9)
Inadequate food access only	126 (90.0)	14 (10.0)	152 (89.4)	18 (10.6)	25 (14.7)	145 (85.3)
High socioeconomic disadvantage only	319 (63.3)	185 (36.7)	498 (65.4)	263 (34.6)	376 (50.3)	372 (49.7)
Low walkability & inadequate food access	1271 (90.3)	137 (9.7)	1402 (84.6)	255 (15.4)	279 (16.9)	1369 (83.1)
**Two neighborhood‐level SDOH**						
High socioeconomic disadvantage & low walkability	559 (59.4)	382 (40.6)	860 (60.4)	565 (39.6)	853 (60.5)	557 (39.5)
High socioeconomic disadvantage & inadequate food access	36 (65.5)	19 (34.5)	47(57.3)	35 (42.7)	39 (48.9)	41 (51.3)
**Three neighborhood‐level SDOH**						
High socioeconomic disadvantage & low walkability & inadequate food access	190 (60.7)	123 (39.3)	229 (56.4)	177 (43.6)	221 (54.7)	183 (45.3)

*Note*: Data are *n* (%) unless otherwise specified.

^a^ Chi‐square tests were used to examine differences in the distribution of iSDOH measures by nSDOH, *p* < 0.001 for all.

When any two nSDOH were present, the proportions of individuals with iSDOH were generally higher compared with when only one nSDOH was present. Adverse iSDOH were most frequent among those living in a community with all three adverse nSDOH compared to those living in communities with any two adverse nSDOH. The frequencies of iSDOH measures (household income, education, and Medicaid status) were significantly higher when compared between communities with all three versus any two versus only one adverse nSDOH measure present (*p* < 0.001 for all).

## 4 DISCUSSION

We found that most nulliparous pregnant individuals lived in a community with one adverse nSDOH (52%), while 43% lived in community with two adverse nSDOH, and 6% lived in a community with all three adverse nSDOH. Adverse iSDOH increased in frequency as individuals lived in communities with a greater number of adverse nSDOH. Finally, there was significant variation in iSDOH factors depending upon the combination of nSDOH that existed for an individual. Namely, individuals who experienced the lowest frequency of iSDOH lived in communities affected by varying degrees of nSDOH.

While many studies have documented the associations of adverse iSDOH with APOs, more recent studies have begun to examine associations between nSDOH and APOs. Prior studies using data from this cohort have found that neighborhood socioeconomic disadvantage as measured by the ADI is associated with abnormal fetal growth, stillbirth, maternal group B streptococcus colonization, postpartum readmission, and the risk of maternal cardiovascular disease in the postpartum period [16, 27, 28, 29, 30]. Among pregnant individuals living with pregestational diabetes, higher socioeconomic disadvantage, low walkability, and inadequate food access are each associated with a lower likelihood of achieving glycemic control [13, 14, 15]. With regard to the relationship between nSDOH and iSDOH, inadequate food access is associated with poorer individual diet quality in early pregnancy [31]. The current analysis builds on these prior studies by exploring the potential for intersectionality among multiple iSDOH and nSDOH domains.

A recent systematic review commissioned by the National Institutes of Health's (NIH) Office of Disease Prevention highlighted the need to identify “themes and patterns at the population level that suggest opportunities for strategy or treatment interventions to address social and structural determinants of health in pregnancy” [18]. Multilevel interventions that influence health at more than one level, such as at both the individual and neighborhood levels, could have a significant public health impact, particularly among disenfranchised pregnant populations [20]. Prior trials outside of pregnancy suggest that addressing individual SDOH factors in isolation does not necessarily improve health outcomes [32, 33, 34, 35]. The neighborhood is a primary contributor to health and well‐being, and structural interventions that address neighborhood‐based social and structural determinants will be necessary to achieve health equity [36]. Whether the varying relationships between iSDOH and nSDOH are differentially associated with risk of APOs remains to be studied.

These findings suggest that iSDOH, such as low household income, lower educational attainment, and Medicaid insurance, are not experienced to the same degree among individuals living in communities with different patterns of nSDOH. There is also variation in the extent to which individuals are exposed to the three community‐level nSDOH factors. Whether multilevel interventions that address such relationships between iSDOH and nSDOH are more efficacious to improve adverse pregnancy outcomes and patient‐reported outcomes in pregnancy requires further study. Future approaches that identify and classify the multifaceted nature of SDOH as they relate to pregnancy outcomes are needed. These data can inform the development of a polysocial risk score that integrates multiple SDOH factors into a single metric or index [37]. A polysocial risk score has recently been developed in relation to cardiovascular disease outcomes [38, 39]; however, such an integrative approach to measuring SDOH should be studied in relation to adverse pregnancy outcomes [40]. Such data could then inform the development of multicomponent interventions that address specific SDOH factors to improve pregnancy outcomes.

A strength of this secondary analysis is its novel use of three previously validated adverse nSDOH to determine their relationship to three adverse iSDOH. This study also used data from a diverse cohort of nulliparous individuals from across the US, which makes these results generalizable to a wider population of pregnant individuals.

There are limitations to note. These data are now over a decade old; however, it is unlikely that the variation between SDOH factors in pregnancy identified in this study has been eliminated. The current analysis was a cross‐sectional analysis wherein iSDOH and nSDOH were assessed concurrently in early pregnancy. Thus, how relationships between iSDOH and nSDOH change over time remains uncertain. Next, it is possible that nSDOH could not be reliably ascertained for some individuals because they lived with unstable housing or in a transient setting. It is likely these individuals would have had greater exposure to adverse SDOH and would be more likely to experience adverse health outcomes. The current study assessed only three iSDOH and three nSDOH and there are additional measures across other SDOH domains that were not assessed. Future analyses may want to employ assessment of other iSDOH and nSDOH domains, such as the five domains emphasized by Healthy People 2030 (economic stability, education access and quality, health care access and quality, neighborhood and built environment, and social and community context) [4].

## 5 CONCLUSION

Among nulliparous individuals, the frequency of iSDOH measures was generally higher with more nSDOH measures, but there were varying patterns of overlap between nSDOH and iSDOH measures. These data emphasize the importance of a multidimensional approach to assessing SDOH risk with several studies confirming the intersectional nature of social determinants outside of pregnancy.

## CONFLICT OF INTEREST STATEMENT

The authors declare no conflicts of interest.

## DISCLAIMER

The views expressed in this article are those of the author and do not reflect the official policy or position of the United States Air Force, Department of Defense, or the U.S. Government.
